# Possible Drug–Radiopharmaceutical Interaction in ^99m^Tc-Sestamibi Parathyroid Imaging

**DOI:** 10.3390/pharmacy13050140

**Published:** 2025-10-01

**Authors:** Tracia-Gay Kennedy-Dixon, Mellanie-Anne Didier, Keisha Allen-Dougan, Peter Glegg, Maxine Gossell-Williams

**Affiliations:** 1 Faculty of Medical Sciences, University of the West Indies, Mona Campus, Kingston 7, Jamaica; 2 Nuclear Medicine Division, Department of Diagnostic & Interventional Radiology, University Hospital of the West Indies, Mona Campus, Kingston 7, Jamaica; 3 Office of the Principal, University of the West Indies, Mona Campus, Kingston 7, Jamaica

**Keywords:** drug-radiopharmaceutical interaction, Technetium-99m Sestamibi, false-negative scan, parathyroid scintigraphy, nuclear pharmacy, Jamaica

## Abstract

Drug–radiopharmaceutical interactions can significantly alter radiotracer biodistribution, complicating diagnostic accuracy. This case report describes a 64-year-old male who underwent a Technetium-99m-methoxyisobutyl isonitrile (^99m^Tc-MIBI) parathyroid scan for suspected primary hyperparathyroidism. Initially, the patient was asked to discontinue his medications for his chronic illnesses for 24 h prior to the scan. However, the images revealed significantly reduced counts/tracer uptake in the thyroid, parathyroid and cardiac tissues in both the early and delayed phases. After a detailed review of his medication profile, it was postulated that there were potential interactions involving multiple P-glycoprotein (P-gp) substrates with specific emphasis on amlodipine, atorvastatin and telmisartan. The patient was advised to discontinue all medications for 72 h prior to the date of a repeat scan which was scheduled for two weeks after his initial scan. The repeat scan successfully detected a small focus of marked tracer retention in the left inferior parathyroid bed, suggestive of a small parathyroid adenoma. Post-surgery, the focus identified on the scan was removed and histologically confirmed to be a parathyroid adenoma. This is the first report of its kind among nuclear medicine patients in Jamaica. It highlights the importance of reviewing medication history prior to nuclear imaging, particularly when using radiotracers affected by P-gp mechanisms. This is crucial for mitigating against false-negative results, thus ensuring accurate diagnosis and appropriate clinical management.

## 1. Introduction

Substantial evidence has been provided to support the concept that the biodistribution of radiopharmaceuticals (RPs) may be altered by the presence of a conventional drug [1]. A drug–radiopharmaceutical interaction results when a conventional drug interacts with the RP, resulting in imaging variations such as decreased labelling efficiency, false positives (results confirming the condition that was tested for, but it is not present), false negatives (results confirming the condition that was tested for is not present, but it is actually present) and increased tissue/organ uptake [2]. These interactions are a crucial consideration in nuclear medicine, as they can impact the efficacy and safety of diagnostic and therapeutic procedures. These interactions can occur through various mechanisms, including pharmacokinetic and pharmacodynamic effects, and can be influenced by factors such as the specific radiopharmaceutical, the drug in question and patient-specific characteristics.

The drug transporter P-glycoprotein (P-gp) is present in many organs and determines the uptake and efflux of a range of drugs. It belongs to the adenosine 5′-triphosphate (ATP)–binding cassette (ABC) superfamily and functions as a transmembrane efflux pump, transporting its substrates from inside to outside the cell [3]. P-glycoprotein is a potential mediator of drug–drug and drug–radiopharmaceutical interactions, as drugs which induce or inhibit the transporter can interact with other drugs that are also handled by the pump. A search of the literature has revealed up to 403 drugs that are substrates, 344 inhibitors and 64 inducers of P-gp, of which quite a number are noted to be both substrate and inhibitor/inducer. This wide spectrum renders P-gp as an important determinant of drug pharmacokinetics and potential drug interactions [4].

In the context of nuclear medicine, P-gp has been implicated in influencing the uptake and efflux of certain radiopharmaceuticals, including Technetium-99m methoxyisobutyl isonitrile (^99m^Tc-MIBI), a commonly used agent for myocardial perfusion imaging and parathyroid adenoma detection. Research has demonstrated that ^99m^Tc-MIBI is a substrate of P-gp, suggesting that variations in P-gp expression or function could impact the biodistribution and diagnostic utility of this radiopharmaceutical. Drug–radiopharmaceutical interactions of this nature have the potential to affect imaging outcomes and clinical interpretations [5]. As such, elucidating the role of P-gp in drug–radiopharmaceutical interactions is crucial for optimizing imaging protocols, predicting potential interactions and ensuring accurate diagnostic assessments in patients receiving multiple medications.

The overall prevalence of drug–radiopharmaceutical interactions is unknown [6]. Considerable work therefore needs to be conducted to ascertain the rate of altered biodistribution due to these interactions, as well as the mechanisms by which they occur. This report seeks to assess the factors that may have contributed to the occurrence of a possible drug–radiopharmaceutical interaction in a patient for ^99m^Tc-MIBI parathyroid scan. The report seeks to contribute to the detection and prevention of future interactions of this nature.

## 2. Case Presentation

A 64-year-old male patient presented for a nuclear parathyroid scan to confirm a diagnosis of primary hyperparathyroidism, having had hypercalcemia and elevated parathyroid hormone levels (Table 1). His past medical history included longstanding hypertension of approximately 30 years duration that had been managed pharmacologically with amlodipine, telmisartan and hydralazine. He also had a documented history of stage five chronic kidney disease (CKD) for the past five years, bilateral knee osteoarthritis, gout, benign prostatic hyperplasia and hypertensive heart disease diagnosed three years prior. He was prescribed carvedilol along with atorvastatin and aspirin as adjunctive therapy for his heart condition.

He had a surgical history of bilateral inguinal hernia repair within the past three months prior to his scan. Six days post-surgery, he was admitted to hospital having experienced a prolonged state of confusion which rendered him disoriented and unable to carry out his usual basic daily functions. Relatives also noted that he had been forgetful for the past month. An MRI of the brain which was performed two days prior to admission revealed moderate atrophy, as well as severe chronic microvascular ischemic changes with multiple chronic infarcts. A CT scan of the brain performed on the day of admission revealed no acute intracranial event but showed sub-acute right frontal lobe ischemic infarct and white matter hypoattenuation, which likely represents small vessel ischemia. He was discharged four days later and was prescribed vinpocetine and donepezil. His full medication profile is listed in Table 2.

Based on institutional protocol, the patient was advised to discontinue all medications for a period of 24 h prior to the nuclear parathyroid scan. On scan day, the sestamibi kit (Bacon Laboratories, Uruguay 136, Buenos Aires, Argentina) was reconstituted with freshly eluted ^99m^Tc sodium pertechnetate from a Lantheus Medical Imaging^®^ TechneLite^®^ Technetium (Tc99m) Generator. The radiopharmaceutical was prepared according to the manufacturer’s instructions, that is, boiling for 10 min followed by cooling for 15 min before administration. The standard protocol for dual-phase parathyroid imaging was utilized with a double-headed Siemens Single Photon Emission Computed Tomography/Computed Tomography (SPECT/CT) gamma camera, Model 10275009, Siemens Medical Solutions, Hoffman Estates, IL, USA, 2018; 750 Megabecquerels (MBq) of ^99m^Tc-MIBI was administered intravenously and the early phase scan was performed 20 min post-injection, while the delayed phase scan was performed 2 h post-injection.

**Figure 1:**
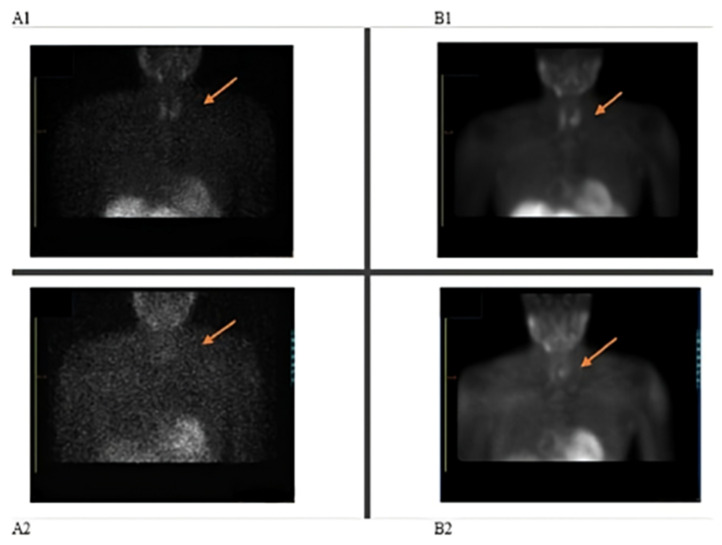
Parathyroid imaging: (**A1**) Initial scan-early parathyroid, (**A2**) initial scan-late parathyroid showing non-localization of parathyroid adenoma; (**B1**) repeat scan-early parathyroid, (**B2**) repeat scan-late parathyroid showing parathyroid adenoma localization.

Unexpectedly, the parathyroid scan demonstrated poor tracer uptake in both the early and delayed phases, as was evidenced by compromised contrast on the acquired images. Subsequent application of attenuation correction, coupled with optimization of reconstruction parameters, led to notable improvements in image quality (Figure 1(A1,A2)). Notably, other patients who received doses from the same radiopharmaceutical kit exhibited normal uptake patterns in these tissues, suggesting a patient-specific issue rather than a problem with the radiotracer batch. Further investigation was warranted to determine the cause of the reduced uptake and to ensure accurate diagnosis. The decision was made by the Nuclear Medicine team to repeat the scan two weeks later, with an adjustment to the patient’s medication protocol. Based on the literature, a clinical decision was made for the patient to discontinue all medications for 72 h prior to the date of the rescheduled scan.

The repeat scan results revealed a small focus of marked tracer retention in the left inferior parathyroid bed, suggestive of a small parathyroid adenoma measuring ~0.7 × 0.6 cm (axial plane) (Figure 1(B1,B2)). Post-surgery the focus identified on the scan was removed and histologically confirmed to be a parathyroid adenoma. Comparative analysis of count statistics between the initial and repeat scans revealed reduced uptake in the initial examination, suggesting factors influencing radiopharmaceutical accumulation.

## 3. Discussion

### 3.1. False Negative ^99m^Tc-MIBI Parathyroid Scan

The sensitivity of ^99m^Tc-MIBI in parathyroid scintigraphy is reported to be between 67% and 86% in some studies [9,10], which has subsequently resulted in false-negative reports from case reports and parathyroid studies conducted in nuclear medicine facilities globally [11,12,13]. False-negative results may impact the course of treatment and the clinical outcome of patients with primary hyperparathyroidism [14] and, therefore, extensive research has been ongoing into the factors that contribute to a false-negative ^99m^Tc-MIBI parathyroid scan [15].

A number of factors have been theorized as causative agents for this phenomenon, of which are serum calcium and parathyroid hormone levels, the size of the overactive parathyroid gland, proportion of chief versus oxyphilic cells, mitochondrial content and p-glycoprotein (P-gp) expression [10,15,16]. Studies have revealed that the high expression of the drug metabolism transporter P-gp in normal parathyroid cells has been postulated to result in negative uptake of ^99m^Tc-MIBI, compared to the low or lack of expression of the transporter in hyperfunctioning cells, which results in accumulation of the radiopharmaceutical [17]. However, there have been instances of negative ^99m^Tc-MIBI imaging in patients with parathyroid adenomas, which revealed positive P-gp expression on the basis of immunohistochemistry [11,17]. In one such study, it was concluded that the P-gp expression may have an important role to play in the false-negative results of ^99m^Tc-MIBI parathyroid scintigraphy in the localization of parathyroid adenomas [11].

### 3.2. P-Glycoprotein

Of the fourteen drugs that the patient in this report was prescribed, five have been classified as a P-gp substate, inhibitor or inducer (Table 2), while ^99m^Tc-MIBI has been classified as a substrate [4,7]. A negative image of ^99m^Tc-MIBI in the parathyroid glands did not immediately warrant alarm; however, the reduced uptake in the patient’s thyroid and cardiac tissues sparked the need for investigation. This was compounded by the fact that doses from the same ^99m^Tc-MIBI kit were administered to other patients on the day for both parathyroid and myocardial perfusion imaging, with uptake noted. A review of the concomitant medications for the other patients who received doses from the identical ^99m^Tc-MIBI kit was conducted, and it has been determined that they were not taking any of the medications used by the index patient. The absence of similar uptake variations in the other scanned patients, coupled with the use of the same kit, supports the theory that the observed alterations in the reported patient likely relate to patient-specific factors. The researchers therefore hypothesize that the lack of radiopharmaceutical uptake in the presenting patient may have been due to the following:

- High expression of P-gp in parathyroid and cardiac cells;
- Increased function of P-gp in parathyroid and cardiac cells;
- Alteration of the pharmacokinetics of ^99m^Tc-MIBI due to the presence of competing substrates, inhibitors and inducers of P-gp.

The presence of the dihydropyridine calcium channel blocker (CCB) amlodipine in the patient’s medication profile is of interest in this investigation. Friedman et al. in their study demonstrated that CCBs reduce scan sensitivity in patients with primary hyperparathyroidism possibly by decreasing the radiopharmaceutical uptake, albeit by a yet undiscovered mechanism. The phenomenon, however, has been attributed to the CCB’s potential to influence the cellular efflux of the radiotracer by altering the function of P-gp, leading to increased efflux from the parathyroid adenoma cells. This reduced retention of ^99m^Tc-MIBI would result in a lower signal on the delayed images, potentially leading to a false-negative result or a less intense, equivocal finding [18]. Amlodipine’s dual nature as a substrate and an inhibitor of P-gp suggests the potential for complex interactions. As a substrate, it competes with other P-gp substrates for transport, and as an inhibitor, it reduces the pump’s ability to transport substrates. At face value, this concept should result in increased intracellular retention of ^99m^Tc-MIBI in the presence of amlodipine, as the radiopharmaceutical is also a P-gp substrate. Notwithstanding, due to the concept of competitive inhibition and substrate-dependent modulation, there is a seeming paradox. Although amlodipine may inhibit the overall pump function, it may also outcompete ^99m^Tc-MIBI for the binding site on the P-gp transporter, thereby increasing its efflux. This phenomenon results in a net decrease in the radiopharmaceutical’s retention in parathyroid adenoma cells [19].

### 3.3. Drug Pharmacokinetics and Pharmacodynamics

An assessment of the half-lives of the prescribed medications for this patient resulted in the postulation that any drug with a half-life of ≤12 h was less likely to have impacted the distribution of the ^99m^Tc-MIBI during the initial scan. A discontinuation of the pharmaceuticals for a period of 24 h prior to the scan would result in trace serum concentrations at the time of radiopharmaceutical administration. The patient was instructed to discontinue all medications for a period of 72 h prior to the repeat scan; therefore, all drugs with a half-life ≥ 36 h have been postulated to not have an effect on scan two as their serum concentration would be of significance, but with no effect on the radiopharmaceutical due to a positive scan result. By this process of elimination, the remaining drugs, atorvastatin, amlodipine and telmisartan, all of which are listed as both substrates and inhibitors of P-gp, and with half-lives between 14 and 35 h, are postulated to be the source of the possible drug–radiopharmaceutical interaction. Their pharmacokinetic profile suggests that they would have been in the patient’s plasma during scan number one, but eliminated at the time of scan number two. Telmisartan, an angiotensin II receptor blocker, has potential indirect effects on parathyroid scan results. Drugs that block the renin–angiotensin–aldosterone system could, in theory, decrease metabolic activity of parathyroid tissue and, hence, ^99m^Tc-MIBI uptake in a hyperfunctioning parathyroid gland [20,21]. Atorvastatin lowers cholesterol by inhibiting the enzyme HMG-CoA reductase, which is responsible for cholesterol synthesis. Although there is a lack of specific evidence directly linking atorvastatin to ^99m^Tc-MIBI uptake in parathyroid scans, theoretically, there is a link between the statin’s mechanism of action and the radiopharmaceutical’s distribution. Research has shown that this class of drugs interferes with mitochondrial function by reducing the synthesis of coenzyme Q10, which is a key mitochondrial component. A reduction in the number or function of mitochondria in parathyroid adenoma cells may impact uptake of ^99m^Tc-MIBI as its retention is dependent on these cells [22,23]. Future studies are poised to substantiate these hypotheses by the determination of possible mechanisms of action via chemical, pharmacokinetic and radiochemical investigations.

### 3.4. Other Variables Affecting ^99m^Tc-MIBI Uptake

Additional factors that may influence radiotracer uptake and distribution include renal function and metabolic variations. Renal impairment may alter the excretion kinetics of radiopharmaceuticals, potentially affecting biodistribution and image interpretation. Research suggests that patients with chronic renal failure may have increased expression of P-gp in their parathyroid glands. This upregulation may result in false-negative scan results [24]. Metabolic variations such as hypercalcemia may modulate radiotracer accumulation. The extent of hypercalcemia may impact ^99m^Tc-MIBI imaging results, as was concluded by Dy et al. in their study, which compared patients with negative scans to those with localization using factors such as laboratory, operative and pathological findings. Results showed that patients in the negative MIBI group had a significantly lower level than those of the positive group. Despite the majority of patients with parathyroid disease presenting with hypercalcemia, those with false-negative scans exhibited relatively lower calcium levels than their counterparts with true-positive scans [25]. Notably, this patient presented with calcium levels of 2.57 mmol/L (normal range 2.06–2.54), which is interpreted as near normal. These factors underscore the importance of considering patient-specific physiological and pathological states when interpreting images as they can contribute to variability in radiotracer distribution and image quality.

### 3.5. Practical Recommendations

This case report contributes to the growing body of literature on drug–radiopharmaceutical interactions, specifically highlighting potential influences of commonly prescribed cardiovascular medications such as amlodipine on ^99m^Tc-MIBI uptake. Aligning with studies demonstrating P-gp-mediated effects on radiotracer distribution, this report is of clinical relevance in highlighting the importance of medication histories in nuclear medicine departments globally. Practical recommendations for nuclear medicine teams include the following:

1. Obtaining detailed medication profiles focusing on P-gp substrates and inhibitors.
2. Considering potential drug interactions when interpreting atypical ^99m^Tc—MIBI uptake patterns in nuclear imaging.
3. Heightened awareness of possible imaging alterations in patients on medications known to modulate transporter activity.

### 3.6. Limitations

The study has several limitations. Firstly, the patient’s laboratory values prior to the nuclear parathyroid scan were available for analysis, providing insight into his biochemical profile at the time of imaging. However, post-scan laboratory data was unavailable, limiting the ability to assess potential changes in relevant parameters post-^99m^Tc-MIBI scan. Consequently, the analysis is restricted to pre-scan data, and correlations between scan outcomes and subsequent laboratory changes could not be evaluated. Secondly, resource limitations precluded comprehensive quality control testing of the radiopharmaceutical to formally exclude alternative explanations such as kit-related technical errors. Nonetheless, patients who received doses that were prepared from the same radiopharmaceutical kit exhibited typical, unremarkable scan patters. This comparative outcome with concurrent patients suggests that the radiopharmaceutical preparation and overall technical process were adequate for imaging purposes. The comparative patient outcomes, therefore, do provide important contextual insight. Thirdly, the absence of pharmacokinetic modelling or molecular expression analyses as a part of this investigation limits direct substantiation of P-gp activity or expression as a contributor to the observed alterations in ^99m^Tc-MIBI uptake. The current observations therefore serve as a basis for hypothesis generation regarding P-gp’s influence on the imaging results. Future studies would benefit from direct experimental or pharmacological investigations.

## 4. Conclusions

This report explored potential drug–radiopharmaceutical interactions, focusing on a case exhibiting altered uptake patterns suggestive of P-gp modulation. The observed variations in ^99m^Tc-MIBI uptake, in the context of concomitant medication use, underscores the plausibility of pharmacological influences on radiopharmaceutical kinetics. This is the first report of its kind among nuclear medicine patients in Jamaica and, by extension, the English-speaking Caribbean. The case highlights the clinical significance of recognizing potential drug–radiopharmaceutical interactions, especially those involving P-gp inhibitor/inducer and substrates. An understanding and appreciation of such interactions in a Jamaican population can inform local clinical practices, drug-prescribing patterns and potentially guide personalized medicine approaches that are relevant to developing countries like Jamaica which have limited resources. This illustrates the need for clinicians to carefully assess medication profiles and patient-specific factors to mitigate false-negative nuclear scan results. Proactive medication management strategies are essential in optimizing diagnostic accuracy and in the prevention of unnecessary repeat imaging, thus ensuring timely and appropriate patient treatment. The expansion and updating of current references to include more information on potential drug–radiopharmaceutical interactions is a step in facilitating this process.

## Figures and Tables

**Table 1:** Laboratory values prior to first nuclear parathyroid scan.

Parameter	Value	Unit	Reference Value
Phosphorus	1.06	mmol/L	0.87–1.45
Calcium	2.57	mmol/L	2.06–2.54
Protein (Total)	75	g/L	60–88
Albumin	45	g/L	35–53
Globulin	30	g/L	20–35
Parathyroid Hormone (PTH)	121	pg/mL	10.4–66.5
Urea	26.8	mg/dL	6–24
Creatinine	324	µmol/L	61.9–114.9

**Table 2:** Prescribed medications, indications for use, their respective half-lives [5] and P-glycoprotein classification [4,7,8].

Drug	Indication for Use	Half-Life (Hours)	P-gp Classification
Vinpocetine	Memory loss	1–2.5	Not classified
Aspirin	Adjunct in heart disease	2–3	Substrate and inducer
Hydralazine	Hypertension	2–8	Not classified
Calcitriol	CKD	5–8	Not classified
Febuxostat	Gout	5–8	Not classified
Carvedilol	Heart disease	6–10	Substrate and inhibitor
Atorvastatin	Adjunct in heart disease	14	Substrate and inhibitor
Telmisartan	Hypertension	24	Substrate and inhibitor
Amlodipine	Hypertension	35	Substrate and inhibitor
Donepezil	Dementia	70	Not classified
Dutasteride and Tamsulosin	BPH	3–5 weeks and 5–7 h, respectively	Not classified
Sevelamer Carbonate	CKD	Unknown	Not classified
Calcium Carbonate	CKD	Unknown	Not classified

CKD–chronic kidney disease, BPH–benign prostatic hyperplasia.

## Data Availability

The data presented in this study are available on request from the corresponding author due to privacy and ethical restrictions.

## Abbreviations

The following abbreviations are used in this manuscript:

^99m^Tc	Technetium-99m
MIBI	Methoxyisobutyl isonitrile
P-gp	P-glycoprotein
RP	Radiopharmaceutical
CKD	Chronic Kidney Disease
MRI	Magnetic Resonance Imaging
CT	Computed Tomography
SPECT	Single Photon Emission Computed Tomography
MBq	Megabecquerel
ATP	Adenosine 5′-triphosphate
ABC	ATP-binding cassette
CCB	Calcium Channel Blockers
